# Genomewide Association Studies for 50 Agronomic Traits in Peanut Using the ‘Reference Set’ Comprising 300 Genotypes from 48 Countries of the Semi-Arid Tropics of the World

**DOI:** 10.1371/journal.pone.0105228

**Published:** 2014-08-20

**Authors:** Manish K. Pandey, Hari D. Upadhyaya, Abhishek Rathore, Vincent Vadez, M. S. Sheshshayee, Manda Sriswathi, Mansee Govil, Ashish Kumar, M. V. C. Gowda, Shivali Sharma, Falalou Hamidou, V. Anil Kumar, Pawan Khera, Ramesh S. Bhat, Aamir W. Khan, Sube Singh, Hongjie Li, Emmanuel Monyo, H. L. Nadaf, Ganapati Mukri, Scott A. Jackson, Baozhu Guo, Xuanqiang Liang, Rajeev K. Varshney

**Affiliations:** 1 International Crops Research Institute for the Semi-Arid Tropics (ICRISAT), Hyderabad, Telangana, India; 2 Department of Crop Physiology, University of Agricultural Sciences (UAS), Bangalore, Karnataka, India; 3 Plant Pathology, Jawahar Lal Nehru Krishi Vishwa Vidyalaya (JNKVV), Jabalpur, Madhya Pradesh, India; 4 Department of Genetics and Plant Breeding, University of Agricultural Sciences (UAS), Dharwad, Karnataka, India; 5 International Crops Research Institute for the Semi-Arid Tropics (ICRISAT), Sahelian Center, Niamey, Niger; 6 Department of Biotechnology, University of Agricultural Sciences (UAS), Dharwad, Karnataka, India; 7 ShanDong Shofine Seed Technology Co Ltd, Jining, Shandong, China; 8 International Crops Research Institute for the Semi-Arid Tropics (ICRISAT), Nairobi, Kenya; 9 Center for Applied Genetic Technologies, The University of Georgia (UGA), Athens, Georgia, United States of America; 10 Crop Protection and Management Research Unit, US Department of Agriculture-Agricultural Research Service (USDA-ARS), Tifton, Georgia, United States of America; 11 Crop Research Institute, Guangdong Academy of Agricultural Sciences (GAAS), Guangzhou, Guangdong, China; National Institute of Plant Genome Research, India

## Abstract

Peanut is an important and nutritious agricultural commodity and a livelihood of many small-holder farmers in the semi-arid tropics (SAT) of world which are facing serious production threats. Integration of genomics tools with on-going genetic improvement approaches is expected to facilitate accelerated development of improved cultivars. Therefore, high-resolution genotyping and multiple season phenotyping data for 50 important agronomic, disease and quality traits were generated on the ‘reference set’ of peanut. This study reports comprehensive analyses of allelic diversity, population structure, linkage disequilibrium (LD) decay and marker-trait association (MTA) in peanut. Distinctness of all the genotypes can be established by using either an unique allele detected by a single SSR or a combination of unique alleles by two or more than two SSR markers. As expected, DArT features (2.0 alleles/locus, 0.125 PIC) showed lower allele frequency and polymorphic information content (PIC) than SSRs (22.21 alleles /locus, 0.715 PIC). Both marker types clearly differentiated the genotypes of diploids from tetraploids. Multi-allelic SSRs identified three sub-groups (K = 3) while the LD simulation trend line based on squared-allele frequency correlations (r^2^) predicted LD decay of 15–20 cM in peanut genome. Detailed analysis identified a total of 524 highly significant MTAs (pvalue >2.1×10–6) with wide phenotypic variance (PV) range (5.81–90.09%) for 36 traits. These MTAs after validation may be deployed in improving biotic resistance, oil/ seed/ nutritional quality, drought tolerance related traits, and yield/ yield components.

## Introduction

Peanut or groundnut (*Arachis hypogaea* L., 2n = 4x = 40) is the mainstay to livelihood of millions of small-holder farmers residing in semi-arid tropic (SAT) regions of the world. This crop is cultivated in 24.6 million ha with the total production of 41.3 million tons and productivity of 1676 kg/ha during 2012. Asia with 11.6 million ha (47.15%) and Africa with 11.7 million ha (47.56%) hold maximum global area. The productivity of Asia (2217 kg/ha) and Africa (929 kg/ha) remained very poor as compared to Americas (3632 kg/ha) [1]. This versatile crop is consumed as cooking oil, fresh/boiled/roasted, as confectionary preparations, flour and peanut butter by human while fresh protein-rich fodder and hay by livestock. In addition, it also plays an important role in making soil healthy through fixing atmospheric nitrogen. Low productivity due to exposure of crops to a range of abiotic (drought, heat) and biotic (foliar diseases, insect pests) stresses especially in Africa and Asia is the major cause for low-income generation to resource-poor farmers. High level aflatoxin contamination is another major concern among the consumers. Further, accumulating adverse impact of drought and heat stress is likely to become even more devastating with inevitable climate change and fast evolving pathogens in unpredictable conditions. Thus, nutrition-rich peanut cultivars possessing genetic resilience for abiotic and biotic stress with enhanced oil/haulm quality and pod yield are required for increased productivity to maintain sustained support to livelihood for millions of poor of SAT region.

Integration of genomics tools with conventional breeding approaches promises to handle the genetic bottlenecks and increase breeding efficiency leading to the rapid development of improved cultivars. In order to deploy genomics-assisted breeding [2], family-based mapping efforts resulted in identification of few quantitative trait loci (QTLs) for simply inherited traits with major phenotypic effect while several QTLs for complex traits with low phenotypic effect [3]. Family-based trait mapping approach has several limitations such as inability to address multiple agronomic traits using single population, time-consuming population development process, use of low density genetic maps, low QTL resolution and overestimation of phenotypic effect of QTLs [4]. Since majority of the agronomically important traits are quantitative in nature, association studies with genomewide marker coverage which allow high resolution mapping of such traits by exploiting historical recombination may enhance the efficiency of candidate gene identification and facilitate genomics-assisted breeding (GAB) for complex traits [5].

In contrast to availability of thousands of most preferred simple sequence repeats (SSRs) in cultivated peanut, very few informative and good quality single nucleotide polymorphisms (SNPs) are available in peanut [3]. The SNPs also pose challenges in interpretation of genotyping data due to polyploidy. Under such circumstances, diversity array technologies (DArTs), therefore, seems to the best high throughput markers. The DArT markers provide the genomewide profiling at a lower cost and in real time in order to conduct comprehensive marker-trait association (MTA) analysis for traits of interest [3], [6]. The peanut ‘mini core collection’ (184 accessions) representing diversity in the peanut ‘core collection’ and entire collection (>14, 000 accessions) was developed at the ICRISAT Genebank [7], . Further, the ‘reference set’ of peanut, was developed based on genotyping of a composite collection (852 genotypes) with 21 SSR markers and phenotyping data for several traits. The ‘reference set’ is comprised of 300 genotypes from 48 countries representing SAT region and include all genotypes of the ‘mini core collection’ [9]. Thus, it represents a very useful material for genetic characterization and high resolution MTA analysis. Multiple season phenotyping data was generated on the ‘reference set’ for many traits under several environments which include diseases resistance, oil/seed/nutritional quality, physiological/drought tolerance related traits, and yield/yield components. There is no comprehensive study done so far in peanut for such economically important traits using dense genotyping and multiple season phenotyping data. In order to fill this research gap, the present study reports the first comprehensive analysis on population structure, linkage disequilibrium (LD) decay and association analysis on a highly diverse germplasm set for several agronomic traits in peanut.

## Results and Discussion

Resource-poor farmers of SAT region with small land holdings need a strong support from genomics and breeding for sustaining their livelihood through increase in their profitability and better health. Limited success could be achieved through genetic improvement approaches in developing improved cultivars performing better under adverse climatic and soil conditions. This situation is going to be further worsened due to fast changing environmental conditions, soil health, limited water and land resources. In addition, inability to identify desirable alleles from germplasm collections and limited use of unique/ rare alleles in breeding programmes led to narrow genetic base in modern cultivars and varieties [10]. Advances in genomics led to the development of genomics resources and tools which upon integration with conventional breeding approaches have shown great potential in developing improved cultivars with desired traits in less time and with more precision in several crop species [11]. Small and fragmented efforts through family-based genetic mapping approaches provided linked markers for few simple traits which are being deployed in marker-assisted breeding [3], [12], [13]. Further, family-based mapping provides only detection of favourable alleles from a limited number of genotypes and thus leaving most of genetic diversity intact. Therefore, the present study is a first comprehensive MTA analysis for identification of molecular markers for a wide range of traits that are of prime importance in developing improved peanut cultivars for SAT regions of Asia and Africa.

The peanut ‘mini core collection’ (184 genotypes) or the ‘reference set’ (300 genotypes) represent the global diversity of about 14, 000 accessions conserved in ICRISAT genebank (Table S1). This germplasm set was well characterized and possess high level of phenotypic variability resilience to biotic/abiotic stresses, pod yield per unit area, oil content, and oil and nutritional quality. Therefore, allelic richness of this germplasm set encouraged us to genotype it with 154 SSRs as well as high throughput DArT arrays with 15,360 features to conduct comprehensive genetic analysis on population structure, allelic/gene diversity, LD-decay and MTA analysis. Data was collected under 467 environments for 50 traits that include five disease resistance traits, six oil and nutritional quality, 27 physiological traits, yield and 11 yield component traits (Table S2).

### Gene/allelic diversity and unique molecular IDs

**Figure 1:**
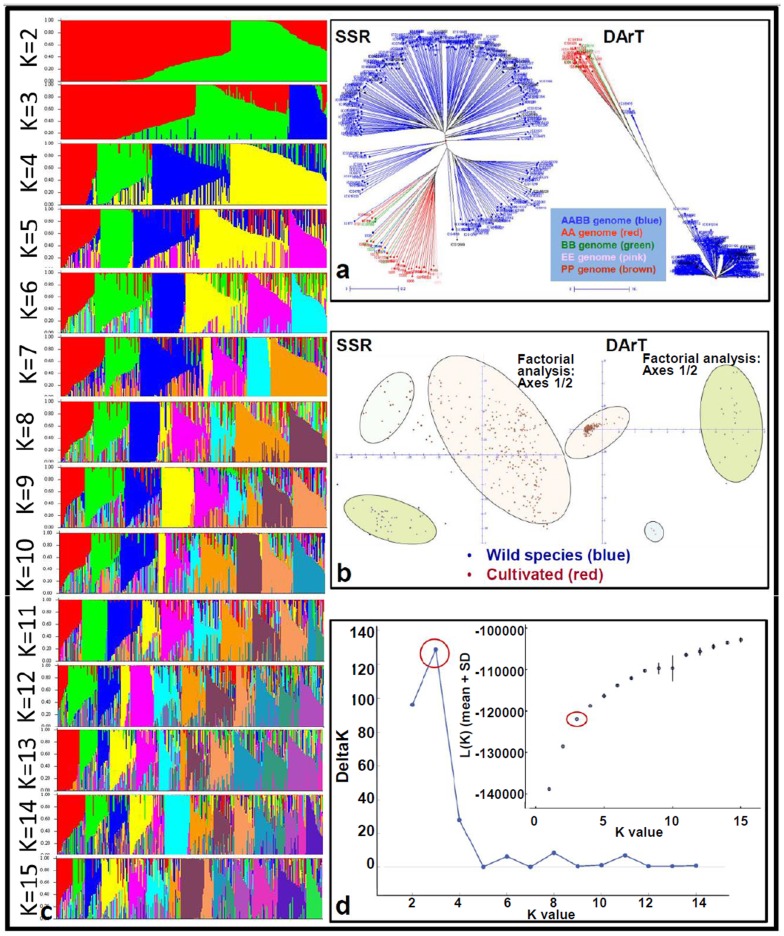
Genetic and population structure of the peanut ‘reference set’. This figure shows (a) grouping of genotypes based on SSR and DArT marker genotyping data, (b) principle co-ordinate analysis (PCoA) based on SSR and DArT marker genotyping data. In the case of SSR as well as DArT based PCoA, cultivated genotypes are clustered in two groups and the wild species genotypes are clustered in one group. (c) the population structure in the reference set at different values of K (K = 1 to K = 15), and (d) presence of three subgroups based on mean Fst values.

Large scale genotyping (>15,000 markers) of the ‘reference set’ provided a great insight in genetic relatedness, identification of most informative markers and other features such as allele number, gene diversity and observed heterozygosity. A total of 9,194 alleles were identified at 4,597 polymorphic DArT loci (Table S3), while 154 SSRs produced a total of 3,420 alleles with an average 22.21 allele per locus (Table S4). Average allele number, gene diversity, heterozygosity and PIC was much higher in SSRs (22.21 alleles per locus, 0.738, 0.079, 0.715) as compared to DArTs (2.00 alleles per locus, 0.174, nil, 0.125). However, the major allele frequency has shown the reverse trend where DArTs (0.901) showed much higher allele frequency for major alleles than SSRs (0.404). The SSR markers also recorded much higher heterozygosity because of being co-dominant in nature while as expected DArTs could not show any observed heterozygosity being dominant in nature. Both the marker systems clearly differentiated the genotypes of different ploidy levels (diploids from tetraploids). Most of the diploid genotypes/accessions (AA, BB, EE, PP) formed a single cluster with a large inter-cluster distance with the clusters of tetraploids (AABB) (Figure 1a and 1b). The SSR markers were found to be superior in differentiating the subgroups of tetraploids while DArTs were superior in resolving tetraploids from the diploids.

Unique molecular IDs for the different accessions are useful in effective germplasm maintenance and plant variety protection. Therefore, multi-allelic data for SSR markers were used to assign unique molecular IDs to all the accessions (Table S5). It is important to mention that 271 (89.2%) out of 304 genotypes analysed has at least one unique allele detected by at least one of 154 SSR markers used. This indicates that these 271 genotypes could be distinguished by using that particular unique allele. By considering unique combination of two alleles generated by at least 2 of 154 SSR markers, 23 (7.6%) more genotypes could be discriminated. The remaining ten (3.2%) genotypes could be well differentiated using unique combination of three or more alleles. Such informative cases were also observed in some crop species such as soybean [14], rapeseed [15], maize [16] and rice [17] but in peanut. These discriminatory markers may be of tremendous use in checking seed impurity, variety identification, germplasm registration and plant variety protection in peanut.

### Population structure and linkage disequilibrium (LD) decay

Multi-allelic SSR markers have always been more effective in revealing the genetic structure of a natural population consisting of diverse genotypes [18]. Therefore, we used multi-allelic data for 154 SSR markers uniformly distributed on the peanut genome to assess genetic architecture and population structure of the peanut ‘reference set’. Upon conducting population structure analysis, it was observed that delta-K declined after K = 3 significantly and continuously, suggesting presence of three sub-groups (Figure 1c and 1d). The assumption for presence of three subgroups was further strengthened through factorial analysis and principal component analysis (Figure 1b). Nevertheless, all the above analysis confirmed presence of three subgroups with high level of admixture within and between subgroups.

**Figure 2:**
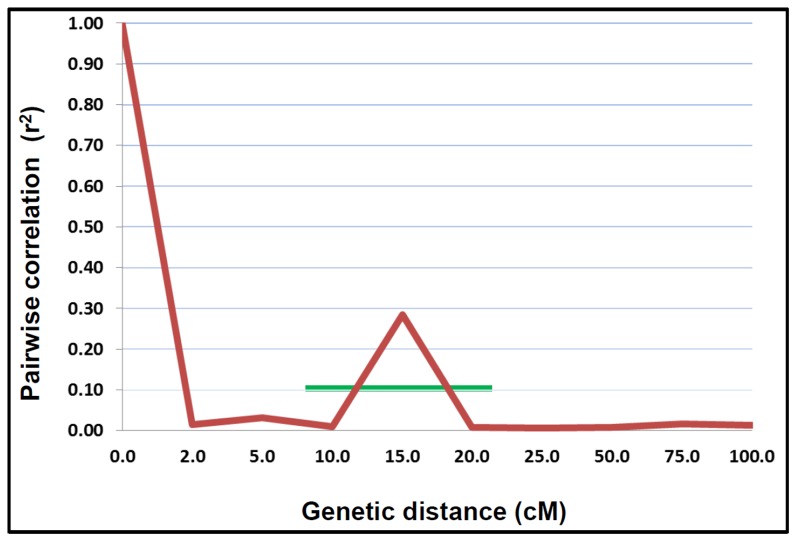
Linkage disequilibrium (LD) decay in the peanut ‘reference set’ based on mapped SSR marker data. The simulation trend line within 20 cM showed that the LD declined to below 0.1 within 20 cM. Hence, the estimated LD decay in the ‘reference set’ is 15.0 cM.

The present study provided most comprehensive insight on population structure and LD decay in a large germplasm set in peanut. Mapping positions for 139 SSR loci mapped onto 20 linkage groups of peanut genome [19] were used for LD estimation. Pairwise LD estimated using the squared-allele frequency correlations (r^2^) was found to decay with the genetic distance of 15 cM (Figure 2). A complete graph for each LG could not be plotted due to availability of less dense mapped markers with uniform genome coverage. Large variation in the magnitude of r^2^ at a given genetic distance was detected reflecting the wide local variation in the extent of LD across the genomic regions. Even after selection and use of multi-allelic SSRs for estimating LD decay in the present study which are supposed to capture more recombination than other marker types during the evolution, the LD decay observed in the present study was low (i.e., high LD) with large LD blocks. Similar large LD blocks were also been detected in many self-pollinated crops such as durum wheat [20], barley [21] and rice [22]. LD blocks of upto 50 cM were detected in durum wheat by using genotyping data for 70 SSRs on a set of 134 genotypes [20], upto 10 cM in barley with 48 SSR markers on 953 cultivated accessions [21], and in rice upto 25 cM with 123 SSR markers on 103 lines [22]. Presence of such large LD blocks in peanut may be due to its high self-pollinating nature, very recent origin with narrow genetic base [23] and relatively small breeding history. The other reason for detecting high LD is the use of limited SSR markers distributed at larger distance in the genome. Once mapped markers distributed at smaller distance in the genome are used, there is a need to re-estimate the LD decay and LD blocks. In fact, the use of relatively larger number of mapped markers as compared to the ones used in the above mentioned studies, showed the faster LD decay in several crops. For instance, 5–10 cM LD decay was observed in wheat by using genotyping data with 518 SNPs and 91 SSRs on 172 elite European winter lines [24], upto 4 cM LD decay was observed in barley with 3072 SNPs on 3840 US breeding germplasm lines [25], and upto 200 kb was observed in rice with 160,000 non-redundant SNPs on 20 accessions [26].

### Marker trait associations (MTAs)

**Figure 3:**
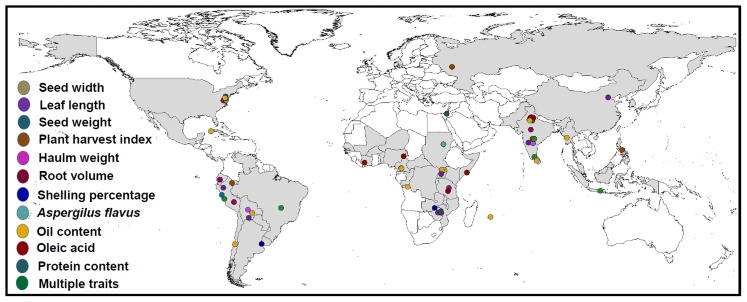
Genomewide distribution of trait-associated markers for different traits. Mapped SSR markers that showed trait association are represented on linkage groups (A01 to A10 and B01 to B10) while unmapped DArT features are assigned to A0 linkage group for representation.

A total of 524 highly significant MTAs for 36 agronomically important traits were identified using most accepted MTA analysis method (P3D mixed linear model with optimum compression) and most stringent multiple test correction method (Bonferroni correction) to filter the false positives (Table 1, Figure 3, Table S6). Phenotypic variance (PV) for these MTAs ranged from low (5.81%) to very high (90.09%). A strong correlation among PV, p-values and F-values has been observed. MTAs detected with high PV for desired agronomically important traits such as disease resistance, oil and nutritional quality, physiological traits, yield and its component traits will foster accelerated genetic enhancement of peanut crop through molecular breeding.

**Table 1:** Marker-trait associations (MTAs) identified for select agronomically important traits.

S. NO	Trait	No. of MTAs	F value range	P value range	PV % range
*Disease resistance traits*					
1	*Aspergillus* (ASP)	1	27.09	9.68E–07	24.69
2	Early leaf spot (ELS)	6	27.03–31.72	4.21E–07–7.59E–08	9.18–10.99
3	Groundnut rosette disease (GRD)	31	5.92–100.19	5.25E–20–7.66E–07	10.25–39.29
4	Late leaf spot (LLS)	1	7.76	1.54E–06	18.1
*Quality and nutritional traits*					
5	Oil content (OC)	25	2.40–42.05	3.18E–10–1.70E–06	5.84–40.37
6	Oleic acid (OLE)	2	5.72	2.47E–06	16.42
7	Oleic/linoleic acid ratio (OLR)	22	4.38–59.98	1.52E–12–1.95E–06	13.67–47.45
8	Protein content (PC)	11	2.99–31.32	8.62E–08–2.14E–06	11.63–36.09
9	Zinc content (ZC)	1	11.29	8.62E–07	15.63
10	Sound mature kernel % (SMK%)	3	9.72–25.24	8.13E–08–2.11E–06	22.21–29.02
*Physiological traits*					
11	Leaf length (LLN)	30	13.75–30.18	6.67E–08–1.26E–06	12.48–21.61
12	Specific leaf area (SLA)	3	23.64–29.29	2.48E–07–2.09E–06	8.72–19.54
13	Total leaf area (TLA)	3	7.33–29.93	1.17E–07–2.03E–06	13.07–15.52
14	Total leaf weight (TLWT)	2	24.89–24.90	1.17E–06–1.20E–06	10.03–10.25
15	Shoot weight (ShWT)	2	23.97–26.78	5.03E–07–1.82E–06	10.38–12.13
16	SPAD chlorophyll meter reading (SCMR)	127	9.00–45.15	1.27E–10–2.12E–06	7.78–18.27
17	Root volume (RTVOL)	1	5.29	6.16E–07	39.59
18	Rate of water loss (RWL)	2	25.75– 31.45	8.28E–07–6.11E–08	11.60 –13.66
19	Haulm weight (HLMWT)	6	24.29–30.35	3.59E–07–1.42E–06	10.16–12.44
20	Harvest index (HI)	41	2.76–77.38	7.35E–17–2.12E–06	5.81–31.80
21	Shelling percentage (ShP)	2	4.29–6.95	8.15E–08–1.99E–06	34.43–36.45
*Yield component traits*					
22	Seed length (SDL)	9	24.37–28.65	2.48E–07– 1.90E–06	11.81–13.29
23	Seed width (SDWD)	3	5.65–25.33	1.82E–07–1.39E–06	14.91–30.09
24	Seed weight (SDWT)	5	5.81–31.54	7.89E–08–2.16E–06	12.73–26.08
25	Pod yield (PYLD)	33	8.68–77.31	4.55E–08–2.16E–06	9.74–37.36
*Traits evaluated under two water regimes (well watered and drought stress)*					
26	Leaf area (LA)_well watered	1	24.09	1.77E–06	9.89
27	Leaf area (LA)_drought stress	1	5.17	8.16E–07	19.84
28	Leaf dry weight (LDW)_drought stress	4	5.10–25.56	8.43E–07–1.24E–06	9.73–19.09
29	SCMR_well watered	16	9.01–41.25	8.05E–10–2.12E–06	9.23–14.31
30	SCMR_drought stress	10	23.81–30.10	1.05E–07–1.91E–06	8.24–12.42
31	Harvest index (HI)_well watered	11	3.22–36.50	2.35E–10–1.40E–06	8.83–39.29
32	Harvest index (HI)_drought stress	36	3.43–38.78	2.97E–10–1.95E–06	8.95–85.40
33	Haulm weight (HLMWT)_well watered	10	4.16–45.67	1.01E–10–2.09E–06	8.93–21.25
34	Haulm weight (HLMWT)_drought stress	10	4.36–53.62	3.56E–12–1.77E–06	9.34–32.26
35	Seed weight (SDWT)_well watered	46	3.49–193.15	7.33E–33–1.93E–06	10.32–88.90
36	Seed weight (WDWT)_drought stress	7	3.95–39.04	3.20E–22–9.03E–08	12.63–90.09
	Total	524	2.4–193.15	7.33E–33–1.32E–06	5.81–90.09

#### Disease resistance

*Aspergillus flavus* (aflatoxin contamination), early leaf spot (ELS), late leaf spot (LLS), rust and groundnut rosette disease (GRD) are among the most devastating diseases in several parts of Asia, Africa and parts of Americas. GRD is endemic in Africa and does not occur in the other continents. A total of 24 season data was collected for these five important diseases at seven locations in 6 countries including India (Bangalore, Dharwad), Malawi, Mali, Senegal, Tanzania and Vietnam (Table S2). Association analysis identified 39 MTAs associated with four of the five diseases with phenotypic variance ranging from 9.18–39.29% (Table 1). Of the 39 MTAs identified, single MTA was for *Aspergillus* (24.69% PV), 31 MTAs for GRD (10.25–39.29% PV), six MTAs for ELS (9.18–10.99% PV), and single MTA for LLS (18.10% PV). The marker associated to one MTA for LLS (Seq1B09) identified in the present study was different than the markers identified in earlier study using biparental populations [27]. Thus, this MTA can be considered as novel QTL/MTA for LLS. Of the four diseases mentioned above, so far no reports are available for identification of associated markers for ELS in peanut. Few reports are available for identification of QTLs for LLS (39 QTLs using family-based mapping approach explaining up to 67.98% PV) [27], [28] and resistance gene analogue mapping (five RGAs explaining up to 43.8% PV) [29]. Similarly, six and eight QTLs were reported for resistance to *Aspergillus flavus* [30] and aphid vector of GRD [31] with PV range of 6.2–22.7% and 1.2–76.1%, respectively. Above results suggest that family-based mapping studies showed much higher predictions of PV for disease resistance traits as compared to present MTA analysis. As all the above four diseases are among the most destructive biotic stresses of peanut in SAT region of Africa and Asia causing serious yield losses, identified MTAs in present study as well as earlier studies may be of great importance for improving disease resistance through use of diverse resistance sources.

#### Quality and nutritional quality

Role of high oil content in increasing profitability along with increased awareness towards health benefits of improved oil and nutritional quality have gained much importance in recent years among producers, consumers and traders. MTA analysis in present study included several oil and nutritional quality traits such as oil content (OC), oleic acid (OLE), oleic / linoleic acid ratio (OLR), protein content (PC), zinc content (ZC), iron content (IC) and sound mature kernels % (SMK%). A total of 32 seasons data on five important traits at six locations in India (Dharwad, Jalgaon, Kawadimatti, Patancheru, Raichur) and Vietnam (Table S2) were generated and used in MTA analysis. A total of 64 MTAs for six quality and nutritional traits were identified with PV ranging from 5.84% (OC) to 47.45% (OLR).

A total of 25 MTAs were detected for OC for which PV ranged from 5.84% (gnPt-714399) to 40.37% (TC4G10) (Table 1). Further, four associated markers namely TC4G10 (40.36% PV), TC11A04 (28.7% PV), Seq7G02 (28.65% PV) and Seq3B05 (22.3% PV) showed high PV and, hence, their deployment may be considered in developing cultivars with high OC. Although, earlier studies identified so far seven QTLs for OC but they showed very low PV (1.5–9.5%) [30], [32], [33]. Therefore, the MTAs identified in the present study will have more impact in increasing OC in peanut. For OLE, only two MTAs linked with single marker Seq5D05 could be detected with 16.42–20.8% PV while 22 MTAs were identified for OLR with PV ranging from 13.7% (gnPt-739706) to 47.45% (GM2480). Two DArT markers (gnPt-739706 and gnPt-736685) with five appearances each and two SSR markers (GM1901, GM2480) with three appearances each showed good consistency. MTAs identified for OLE possessed lower PV than the earlier identified MTA conducted on ‘US-mini core collection’ (53.57% PV) while MTAs identified for OLR were little higher than the PV% of earlier study (42.35%) [34].

A maiden attempt was made here for identifying MTAs for three important nutritional quality traits which resulted in detection of 11 MTAs (11.63–36.1% PV) for PC and single MTA for ZC (15.63% PV) while no MTA for IC (Table 1). In addition to above nutritional quality traits, three MTAs were detected successfully for an important pod quality trait i.e., SMK% (22.2%–29.02%). Although 10 QTLs were reported with low PV (1.5–13.5%) for PC using family-based mapping approaches [30], [33] but so far no QTL/MTA was reported for ZC and SMK%. Thus, the present study reports the first comprehensive analysis for addressing above mentioned oil and nutritional quality traits and provides a glimpse on greater genetic control of these important traits.

#### Physiological traits

The environmental, soil moisture and climatic resilience of a plant depends on the interaction between abiotic stresses and several physiological traits which finally affect the survival and reproduction of crop plants. Some of these traits include Δ^13^C, harvest index (HI), haulm weight (HLMWT), leaf dry weight (LDWT), leaf area (LA), leaf length (LLN), leaf weight (LWT), leaf width (LWD), root / shoot ratio (RSR), rate of water loss (RWL), root length (RTL), root volume (RTVOL), root weight (RWT), shelling percentage (ShP), shoot length (SLN), shoot weight (SWT), specific leaf area (SLA), total leaf area (TLA), total leaf weight (TLWT), SPAD chlorophyll meter reading (SCMR), total dry matter (TDM), TDM/LA, days to flowering (DF), days to maturity (DM), emergence (EMR) and first flowering (FFL). A total of 208 seasons data on these 26 important physiological traits characterised in four countries including India (Bangalore, Dharwad, Durgapura, Jalgaon, Kawadimatti, Patancheru, Raichur), Niger (ICRISAT Sahelian center), Thailand and Vietnam (Table S2) were used in the analysis. In addition to phenotyping data generated under normal conditions i.e., without any stress, few experiments were also conducted under both the conditions (well watered and drought stress).

MTA analysis conducted for the data generated under normal conditions only, identified a total of 219 MTAs for 11 physiological traits namely LLN (30 MTAs, 12.48–21.61% PV), SLA (three MTAs, 8.72–19.54% PV), TLA (three MTAs, 13.07–15.52% PV), TLWT (two MTAs, 10.03–10.25% PV), SCMR (127 MTAs, 7.78%–18.27% PV), RTVOL (single MTA, 39.59% PV), RWL (two MTAs, 11.60–13.66% PV), ShWT (two MTAs, 10.38–12.13% PV), HLMWT (six MTAs, 10.16–12.44% PV), HI (41 MTAs, 5.81%–31.80% PV) and ShP (two MTAs, 34.43–36.45% PV) (Table 1). So far a total of 13 QTLs (3.48–13.29% PV) were reported by earlier studies using a family-based mapping population (TAG 24×ICGV 86031) [35], [36] for SLA while no QTL/MTA was reported so far in peanut for LLN, TLA, TLWT, RWL and RTVOL. Further same population (TAG 24×ICGV 86031) identified a total of 29 QTLs for SCMR (5.72–19.52% PV), 11 QTLs for ShWT (5.03–22.09% PV) and six QTLs for HLMWT (3.78–33.66% PV) [35], [36]. In addition, earlier study also reported three QTLs for HI (6.39–40.10% PV) [19] through family-based mapping approaches while no QTLs/MTAs could be identified for shelling percentage so far in peanut. Thus, MTAs identified in the present study have high significance towards understanding the genetic control of these traits and may facilitate genetic enhancement to provide greater resilience and positive support towards maintaining the physiological balance to peanut crop.

#### Yield and yield component traits

Yield and yield component traits have been the prime target of improvement in all the breeding programmes. Total 12 yield component traits (plant number-PLN, plant height-PHT, pod length-PDLN, pod width-PDWD, pods per plant-PPP, primary branching-PBR, pod weight-PDWT, seed length-SDL, seed width-SDWD, seed weight-SDWT, test weight-TW and pod yield-PYLD) were analysed. A total of 50 MTAs could be identified for four yield component traits with PV ranging from 9.74% (PYLD) to 37.6% (PYLD). Significant MTAs could be identified only for SDL (nine MTAs, 11.81–13.29% PV), SDWD (three MTAs, 14.91–30.09% PV), SDWT (five MTAs, 12.73%–26.08% PV) and pod yield (33 MTAs, 9.74–37.36% PV) (Table 1). One of the associated marker (Seq5D05) with SDWT showed good consistency and appeared thrice (total five MTAs) with stable and high phenotypic variance (24.56%, 24.77%, 26.08%) and hence, is promising. Further, of the 33 MTAs identified for pod yield, four associated markers (gnPt-551105, gnPt-583176, gnPt-583192 and gnPt-583472) have shown consistency by appearing twice each in two environments (YPP_IC01PR and YPP_IC03PR). Interestingly, seven MTAs could be detected with comparatively higher (>20%) PV at Malawi during 2010 with PV ranging from 20.91 to 37.36% (gnPt-584383, gnPt-585177, gnPt-739424, gnPt-584202, gnPt-734871, gnPt-734806 and GM1445) and targeting this region may be of great importance in improving yield. Five QTLs were identified through family-based mapping approach for seed weight (4.18–19.8% PV) [35], [36], [37] while so far no QTL/MTA could be identified for seed length, seed width and pod yield except the present study. The above MTAs will be of great use in improving yield components and pod yield through molecular breeding.

#### MTAs under well watered (WW) and drought stress (DS) conditions

Drought stress has been the most devastating abiotic stress resulting in huge yield loss along with high level of *Aspergillus* infection resulting into aflatoxin contamination of the produce and thus making it unsafe for consumption. Small-holder farmers of SAT regions have been among the most affected with drought stress and hence, continuously posing threat to their health, income and sustainable livelihood. With an objective to detect MTAs for physiological traits affecting drought tolerance significantly for use in GAB, an experiment was conducted under both the conditions (well watered-WW and drought stress-DS) to understand the genetic mechanism and common genomic regions controlling these physiological traits.

A total of 152 MTAs were detected under both the conditions (WW and DS) for six traits namely LA, LDWT, SCMR, HLMWT, HI and SDWT (Table 1). A total of 84 MTA were identified under well watered condition which include single MTA for LA (9.89% PV), SCMR (16 MTAs, 9.23–14.31% PV), HLMWT (10 MTAs, 8.93–21.25% PV), HI (11 MTAs, 8.83–39.29% PV) and SDWT (46 MTAs, 10.32–88.90% PV) (Table 1). No MTA was detected for LDWT under well watered condition. Of the 152 MTAs, 68 MTAs were identified under drought stress condition including LA (single MTA, 19.84% PV), LDWT (4 MTAs, 9.73–9.09% PV), SCMR (10 MTAs, 8.24–12.42% PV), HLMWT (10 MTAs, 9.34–32.26% PV), HI (36 MTAs, 8.95–85.40% PV) and SDWT (7 MTAs, 12.63–90.09% PV). Interestingly, of the 53 MTAs for SDWT, 46 MTAs were identified under well watered condition with PV ranging from 10.32% (gnPt-735510) to a maximum of 88.90% (TC1B02). Eleven markers showed PV more than 50% ranging from a minimum of 53.97% to 88.89% under well watered condition. Of the seven MTAs detected under drought stress condition, PV varied from 12.63% (gnPt-586265) to 90.09% (GM2589). Similarly, of the 47 MTAs detected for HI, five MTAs each under drought stress condition with PV% from 25.4%–85.39% (TC7C06, GM1809, Seq19G07, TC7E04 and GM2589) and well watered conditions showed high PV% i.e., 32.40%–39.29% (GM2638, GM1076, GM1469, GM1609 and GM2350). Thus, the identification of these associated markers with above important traits is of great interest to breeders willing to improve these traits with molecular markers. So far no report is available from such studies wherein the data was collected on both the water regimes on the above traits and hence no such MTAs/QTLs were reported earlier with best of our knowledge.

In the past, only few studies have been conducted on trait mapping for economically important traits in peanut and therefore, very limited information is available on the markers linked to the traits analysed in this study. Furthermore, use of different kind/set of markers in these studies don't allow a possibility to compare results of this study with the previous studies. Nevertheless, some SSR markers used in the present study were also used in the previous linkage mapping based marker-association studies [19], [27], [28], [33], [35], [36]. Of these linked markers, only five markers were found associated in present as well as one of the above mentioned earlier studies. For example, Seq5D05 was found linked to rust resistance in the earlier study [28] and was also found to be associated with oil content, oleic acid, harvest index and seed weight in the present study. Similarly, the marker TC3E05 identified earlier linked to SCMR, haulm weight and total dry weight [19] was found associated with seed weight under drought stress in the present study. The marker S108 and Seq7G02 identified earlier associated with late leaf spot resistance [28] showed association with leaf length, SCMR, oil content and zinc content in the present study. Similarly, the marker TC11A04 reported earlier linked with rust resistance [28] was found associated with protein content, oil content and harvest index in the present study. However, marker TC3E05 showed association with the related traits in the present study (seed weight under drought stress) and the previous study (haulm weight).

### Significant MTAs for molecular breeding

**Figure 4:**
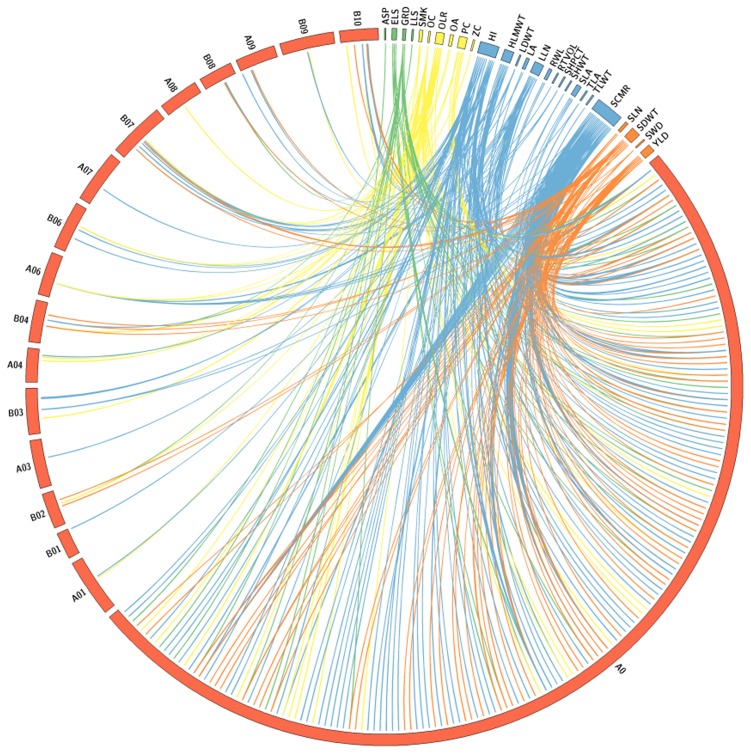
Global distribution of genotypes containing linked-marker allele(s) for different economically important traits in peanut. An attempt has been made to show passport-based geographical distribution of genotypes that had favourable alleles for markers showing association and explaining >20% phenotypic variation for the trait. Genotypes containing favourable alleles for different traits have been represented by circles in different colors.

The main objective of this study was to identify MTAs for agronomical traits of complex nature using diverse panel of genotypes. As a result, a total of 134 MTAs were identified with PV >20% for 15 important traits. A total of 30 significant allele effects for these 15 traits were identified associated with 24 markers showing significant impact on these traits while nine markers were found to be associated with multiple traits (Table 2). Fifty nine genotypes with combination of favourable allele for 11 individual traits as well as for multiple traits were identified (Figure 4). In addition, nine genotypes possessing favourable alleles for multiple traits (Table 3) which might serve as potential donors for improving respective traits. All these associated markers and identified genotypes with favourable alleles can be deployed after validation for improving above mentioned traits through molecular breeding.

**Table 2:** Allelic effect of selected marker-trait associations (MTAs) with ≥20% phenotypic variance for 15 traits.

S. No.	Traits	Season	Marker		PV %	Associated locus	Mean phenotypic value			Locus effect on the trait phenotype
			Name	Type			Genotypes with +ve locus	Population mean	Genotypes with -ve locus	
1	*Aspergilus* infection	Dharwad 2008	gnPt-737044	DArT	24.7	0	1.8	3. 6	3.6	0
2	Groundnut rosette disease (GRD)	Malawi 2010	GM1445	SSR	30.3	265∶265	0.0	95.7	96.1	−97.5
			GM1416	SSR	22.5	92∶92	13.6	95.7	96.0	−86.3
3	Yield under GRD stress	Malawi 2010	GM1445	SSR	37.4	265∶265	745.5	52.8	49.9	739.9
4	Harvest index (HI)	Patancheru 2003R, 2003PR, 2005R, 2005PR	GM2062	SSR	30.7	392∶402	90.8–329.2	45.3–56.0	45.1–54.5	126.1
			GM2062	SSR	30.7	392∶392	65.9–138.1	45.3–56.0	45.2–55.6	17.9
			TC11A04	SSR	26.5	182∶182	63.2–70.2	40.9–47.4	40.8–47.3	22.9
			Seq15C10	SSR	31.8	214∶270	64.5–68.6	40.9–47.4	40.8–47.3	21.7
			Seq19D09	SSR	20.1	262∶262	52.6–60.2	40.9–47.4	40.8–47.4	21.4
			Seq19D09	SSR	20.1	274∶274	45.6–53.3	40.9–47.4	40.8–47.4	18.4
5	Haulm weight (HLMWT)	Niger 2009R	GM2745	SSR	20.4	256∶256	2462.7	1085.3	1079.9	2030.9
			GM2745	SSR	20.4	243∶243	2286.3	1085.3	1080.6	1264.9
			PM419	SSR	20.0	193∶193	2462.7	1085.3	1079.9	1862.5
6	Leaf length (LLN)	Patancheru 2003R, 2003PR, MeanR	S108	SSR	21.6	199∶199	56.8–57.3	51.8–51.9	51.5–51.7	5.2–5.5
7	Root volume (RTVOL)	Bangalore 2010	PM346	SSR	39.6	203∶203	34.9	16.9	16.8	23.2
			PM346	SSR	39.6	206∶206	34.9	16.9	16.9	22.2
8	Seed weight (SDWT)	Patancheru 2009	GM2589	SSR	90.1	314∶324	293.8	39.4	38.4	269.5
			TC1E01	SSR	71.9	247∶247	293.8	39.4	38.4	263.1
			TC3E05	SSR	82.1	304∶304	293.8	39.4	38.4	250.9
			GM1878	SSR	41.7	127∶127	167.4	39.4	38.4	122.7
9	Seed width (SDWD)	Thailand 2004R	GM2745	SSR	30.1	190∶190	3.8	3.6	3.7	2.1
10	Shelling (SH) %	Patancheru 2004PR	GM2531	SSR	34.4	298∶298	71.9	66.1	66.1	8.9
			GM2531	SSR	34.4	302∶302	69.9	66.1	66.1	7.1
			GM2531	SSR	34.4	298∶310	71.3	66.1	66.1	4.8
11	Sound mature kernels (SMK) %	Dharwad 2008	gnPt-550164	DArT	22.2	0.04236	93.9	93.4	87.3	9.1
12	Oil content (OC)	Patancheru 2001R	Seq7G02	SSR	28.6	218∶230	53.8	49.6	49.6	8.9
			Seq7G02	SSR	28.6	220∶224	51.6	49.6	49.6	7.0
		Patancheru 2001R, 2001PR	Seq3B05	SSR	22.5	270∶270	52.0–53.0	49.6	49.6	7.8
			Seq3B05	SSR	22.5	300∶300	50.9–51.5	49.6	49.5–59.5	6.9
13	Oleic acid (OLE)	Patancheru 2009	Seq5D05	SSR	20.8	274∶274	54.9	48.8	48.3	5.2
14	Oleic/linoleic acid ratio (OLR)	Dharwad 2009, MeanR	GM1445	SSR	33.1	247∶247	7.5	1.67	1.6	6.2
			GM2480	SSR	47.4	223∶223	7.5	1.67	1.6	6.1
			GM1901	SSR	37.1	151∶151	7.5	1.67	1.6	6.0
			Seq13E09	SSR	38.2	289∶299	7.5	1.67	1.6	5.6
15	Protein content (PC)	Patancheru 2001R, 2001PR	TC11A04	SSR	36.1	168∶184	20.1–21.0	18.9	18.9	1.9
			TC11A04	SSR	36.1	168∶200	19.8–20.7	18.9	18.9	1.7

**Table 3:** Selected genotypes possessing desirable allelic combination for multiple traits.

Genotypes	Trait	Season	Associated marker	Associated locus (allele 1: allele 2)	Phenotypic value
ICG 14705	Groundnut rosette disease (GRD)	Malawi 2010	GM1445	265∶265	0.0
	Yield under GRD stress	Malawi 2010	GM1445	265∶265	745.5
ICG 13099	Groundnut rosette disease (GRD)	Malawi 2010	GM1416	92∶92	13.6
	Oleic acid (OLE)	Patancheru 2009	Seq5D05	274∶274	54.2
ICG 10890	Harvest index (HI)	Patancheru 2003PR	Seq15C10	214∶270	68.6
	Harvest index (HI)	Patancheru 2005PR	Seq15C10	214∶270	64.4
	Oil content (OC)	Patancheru 2001R	Seq3B05	300∶300	51.2
	Oil content (OC)	Patancheru 2001PR	Seq3B05	300∶300	51.0
ICG 3584	Harvest index (HI)	Patancheru 2003PR	Seq19D09	262∶262	60.2
	Harvest index (HI)	Patancheru 2005PR	Seq19D09	262∶262	52.6
	Shelling (SH) %	Patancheru 2004PR	GM2531	298∶310	71.3
ICG 188	Oil content (OC)	Patancheru 2001R	Seq3B05	300∶300	49.6
	Oil content (OC)	Patancheru 2001PR	Seq3B05	300∶300	53.0
	Protein content (PC)	Patancheru 2001R	TC11A04	168∶200	20.0
	Protein content (PC)	Patancheru 2001PR	TC11A04	168∶200	19.0
ICG 13603	Oil content (OC)	Patancheru 2001R	Seq3B05	300∶300	49.1
	Oil content (OC)	Patancheru 2001PR	Seq3B05	300∶300	49.0
	Protein content (PC)	Patancheru 2001R	TC11A04	168∶184	20.0
	Protein content (PC)	Patancheru 2001PR	TC11A04	168∶184	19.6
ICG 928	Oleic acid (OLE)	Patancheru 2009	Seq5D05	274∶274	54.2
	Seed weight (SDWT)	Patancheru DS2009	GM2589	314∶324	293.8
ICG 2381	Oleic acid (OLE)	Patancheru 2009	Seq5D05	274∶274	54.2
	Oleic/linoleic acid ratio (OLR)	Dharwad 2009	GM1445	247∶247	7.5
ICG 12682	Oleic acid (OLE)	Patancheru 2009	Seq5D05	274∶274	54.2
	Seed weight (SDWT)	Patancheru DS2009	GM2589	314∶324	33.7

## Conclusions

In view of making peanut crop more resilient to stresses with high pod and oil yield and improved oil and nutritional quality, this study is the timeliest and most comprehensive marker-trait association study conducted so far in peanut using thousands of markers and multiple season phenotyping data generated on wide range of economically important traits. Thus, several MTAs detected for many disease resistance, oil content and quality, drought tolerance related (physiological) traits, yield components and yield in the present study based on multiple season phenotyping data will facilitate their improvement through GAB. To achieve this, these MTAs upon validation may be deployed in marker-assisted improvement of peanut leading to development of improved cultivars with higher resilience to drought tolerance and disease resistance, increased yield and, improved oil and nutritional quality. Such improved cultivars will ensure sustainable livelihood to the farmers of SAT regions of Africa and Asia, and better nutritional supply to the consumers' worldwide.

## Materials and Methods

### Plant material and DNA isolation

The peanut ‘reference set’ (300 genotypes) along with four additional elite genotypes was genotyped with 154 SSRs (genomic/genic) spanning complete peanut genome (Table S1). The ‘reference set’ possess representative genotypes from 48 countries including different genomes (AA, BB, EE, EX, PP and AABB). The leaf sample collection, isolation of total genomic DNA following modified CTAB-based method, quantification and quality check of DNA was done as per Cuc et al. [38].

### Genotyping of the ‘reference set’ with SSR markers

A total of 154 SSR markers were used in the present study and details of these markers have been provided in Table S4. Primer pairs for these SSRs were synthesized and PCR reactions were performed in 5 µl volume following a touchdown PCR profile in an ABI thermal cycler (Applied Biosystems, USA) for all markers. SSR genotyping and allele scoring was done as per procedure explained in Cuc et al. [38] and Varshney et al. [35]. PCR master mix was prepared containing ∼5 ng of genomic DNA, 2 picomoles of each primer, 2 mM of each dNTP, 2 mM MgCl_2_, 1X amplification buffer and 0.1 U of Taq DNA polymerase (SibEnzyme, Russia). Primers were amplified using touchdown PCR amplification profile which had initial denaturation step for 3 min at 94°C followed by first 5 cycles of 94°C for 20 sec, 65°C for 20 sec and 72°C for 30 sec, with 1°C decrease in temperature for each cycle, followed by 35 cycles of 94°C for 20 sec with constant annealing temperature (59°C) for 20 sec and 72°C for 30 sec, followed by a final extension for 20 min at 72°C. After getting the amplified PCR products, agarose gel (1.2%) was used for checking the amplification of markers. The PCR products with good amplification were then used for estimating fragment size.

For estimating amplicon length size, amplified PCR products were diluted to 60-100 folds in order to use them for multiplexing SSRs based on their fluorescent labels and amplicon length. SSRs with different labels and allele size ranges were considered together to get good multiplexes. The PCR products (1 µl) with GeneScan 500 LIZ standard (Applied Biosystems) from all the SSRs of a single multiplex were then mixed with formamide (1 µl) in each well. Capillary electrophoresis (ABI 3700 Genetic Analyzer-Applied Biosystems) was then used to analyse amplified products. Result files were then transferred to computer to do allele sizing using GENEMAPPER v4.0 software (Applied Biosystems). In addition, few SSRs which were not amenable to ABI genotyping, PCR products of these markers were analysed on 6% non-denaturing polyacrylamide gels (PAGE) (29∶1 acrylamide/bisacrylamide) and visualized by silver staining.

### Genotyping of the ‘reference set’ with DArT markers

DArT arrays in peanut have been developed by DArT Pty Ltd, Australia in collaboration with ICRISAT (India). The peanut ‘reference set’ has been genotyped with a DArT array consisting of 15,360 features. A total of 4,597 markers/features were polymorphic which were used for population diversity and association study. The detailed method of genotyping is available in the website of (http://www.diversityarrays.com/molecularprincip.html), however, the method is briefly described below. DArT technology consists of several steps such as complexity reduction of the DNA of interest, library creation, microarraying libraries onto glass slides, hybridisation of fluoro-labelled DNA onto slides, scanning of slides for hybridisation signal and data extraction for analysis. The complexity of a DNA sample was reduced to obtain a ‘representation’ of that sample and then variation for that representation is determined which reflect sequence variation. DArT markers detect variations of its presence vs. absence in a genomic ‘representation’ through hybridisation to DArT array consisting of a library from peanut. In this case, earlier a library for peanut representing mixture of genomic ‘representations’ from a pool of individuals covering the genetic diversity of the species is amplified. These fragments were then cloned into a vector that was introduced into *E. coli* to form a library and each colony contained one of the fragments from the genomic ‘representation’. Currently the high-throughput capability of DArT is based on a microarray platform and, selections of clones from the library are arranged into a plate format (usually 384-well plates) after library creation. The fragments within the library were amplified and spotted onto glass slides using a microarrayer to form a genotyping array. After washing and processing of these hybridised slides to remove unbound labelled DNA, the slides are then scanned using a scanner to detect fluorescent signal emitted from the hybridised fragments. Finally, the result from each fluorescent channel is recorded and the data from the scanned images was extracted and analysed using the DArTsoft software and the information is managed by the DArTdb Laboratory Information Management System.

### Phenotyping of the ‘reference set’

The peanut ‘mini core collection’ is a subset of the ‘reference set’ and either complete ‘reference set’ or ‘mini core collection’ was characterised for a total of 50 agronomic traits (Table S2). These traits include disease resistance (*Aspergillus*, early leaf spot, groundnut rosette disease, late leaf spot, rust resistance), oil and nutritional quality (oil content, oleic acid, oleic / linoleic acid ratio, protein content, zinc content, iron content), physiological traits (Δ^13^C, sound mature kernel percentage, harvest index, haulm weight, leaf dry weight, leaf area, leaf length, leaf weight, leaf width, root / shoot ratio, rate of water loss, root length, root volume, root weight, shelling percentage, shoot length, shoot weight, specific leaf area, total leaf area, total leaf weight, SPAD chlorophyll meter readings, total dry matter, total dry mass/leaf area, days to flowering, days to maturity, emergence, first flowering), yield and its components traits (plant number, plant height, pod length, pod width, pods per plant, primary branching, pod weight, seed length, seed weight, seed width, test weight, yield per plant, plot yield). Evaluation and characterization was done in a total of 467 environments at 157 locations (14 locations used for several environments). These 14 locations from eight countries included Patancheru, Bangalore, Dharwad, Raichur, Jalgaon, Durgapura and Coimbatore in India, Malawi, Mali, Senegal, Tanzania, Niger (ICRISAT Sahelian Centre), Vietnam and Thailand. The details on standard procedure for phenotyping are explained in several earlier published literature [39], [40], [41], [42], [43].

### Diversity and cluster analyses

The polymorphism information content (PIC), major allele frequency, number for observations, availability and gene diversity were calculated using the software PowerMarker ver. 3.25 [44] and DARwin ver. 5.0.158 [45].

### Population structure and linkage disequilibrium analysis

The genetic structure and number of subgroups of this germplasm set was estimated using the model-based Bayesian clustering method implemented in STRUCTURE software version 2.1 [46]. This approach best uses multi-locus genotypic data (i.e., in case of SSRs) without prior knowledge of their population affinities and assumes loci in Hardy-Weinberg equilibrium in order to assign individuals to clusters/groups (K). STRUCTURE analysis and subgrouping were decided following Kulwal et al. [47]. Admixture model with correlated allele frequencies was used to estimate each of the K clusters from 1 to 20 (hypothetical number of subgroups) for each accession along with the percentage of its genome derived from each cluster. We set other parameters at higher level to achieve reliable subgrouping such as length of burning period of 1,00,000 and number of MCMC (Markov Chain Monte Carlo) replications after burning of 2,00,000. In order to get consistent and reliable subgroupings, each K was repeated five times i.e., iterations/replications. As rare alleles induce large variances, only markers with a minor allele frequency of at least 0.05 were included in the analysis.

Estimated likelihood values [LnP(D)], log likelihood of the observed genotype distribution in K clusters obtained from STRUCTURE runs against K was used to predict the most probable number of subgroups in the population. The delta-K value best describes the population structure based on the criteria of maximizing the log probability of data or in other words the value at which LnP(D) reaches a plateau and hence, delta-K was calculated in order to have appropriate subgroups in this population.

Linkage disequilibrium (LD), which provides an estimate for number of markers required for conducting genetic/QTL mapping and GWAS, sometimes influenced by population structure and subgrouping derived based on the demographic and breeding history of the accessions included in the germplasm set. Genomewide LD in the present set was estimated by pair-wise comparisons among the genome anchored 139 SSR markers. Squared allele frequency correlations (r^2^) between the pairs of loci were used for calculating LD [48]. Since the number of mapped loci were not sufficient to estimate LD for each linkage group, average LD decay in the whole genome among the panel with r^2^ values were plotted against the genetic distance (cM) between markers.

### Marker-trait association analysis

In order to conduct precise marker-trait association analysis, population structure and Q values from the software STRUCTURE while principal components (PCs) obtained from TASSEL were used as covariates during MLM analysis. Further among different options available within MLM, the widely adapted approach called “optimum levels of compression in combination with P3D” for variance component estimation was used for association analysis. For MLM analysis, marker-based kinship matrix (K) obtained using TASSEL was used along with the Q matrix to correct for both family and population structure and the phenotypic variation explained (r^2^) by the marker is reported [47], [49].

### Correction of false discovery rate

Of the several methods suggested to correct false positive in association analysis even keeping stringent p-value benchmark, the most stringent correction method called “Bonferroni Correction” was used in the present analysis. The threshold was found to be 2.1×10^−6^ at a significance level of 1% after Bonferroni multiple test correction (0.01/4751). The denominator in the Bonferroni correction is the total number of markers tested.

## Supporting Information

Table S1 **Details on the peanut ‘reference set’ in terms of genome type, origin and biological status.** This table contains list of genotypes used in this study. It also contains information on species, market types (runner or bunch), genome information, geographical region and biological status for all the genotypes.
(XLS)

Table S2 **Summary on phenotyping data collected on the peanut ‘reference set’.** This table contains information on names of locations where phenotyping experiment was conducted. It also contains information for each trait about number of environments and locations.
(XLS)

Table S3 **Major allele frequency, gene diversity and PIC content of 4597 DArT loci assayed on the peanut ‘reference set’.** This table contains information on major allele frequencies estimated, number of alleles scored, gene diversity calculated and polymorphic information content (PIC) calculated for each polymorphic DArT marker.
(XLS)

Table S4 **Major allele frequency, gene diversity and PIC content of 154 SSR loci screened on the peanut ‘reference set’.** This table contains information on major allele frequencies estimated, number of alleles scored, gene diversity calculated, heterozygosity observed and polymorphic information content (PIC) calculated for each polymorphic SSR marker. The information on sequence and source of development of these SSRs have also been provided in this table.
(XLS)

Table S5 **Marker allele unique to the genotype identified for all the genotypes of the peanut ‘reference set’.** This table contains information on unique allele identified for selected SSR markers which can differentiate each genotype from the other genotypes. Majority of the genotypes could be identified by unique allele detected by a single marker and the remaining genotypes could be discriminated by using a combination of unique alleles for two or more than two SSR markers. Alleles highlighted with green colour indicate uniqueness to a single genotype. Dark orange colour indicates that this allele is present only in two genotypes; blue colour alleles are present in three genotypes while purple colour indicates presence in four or five genotypes.
(XLSX)

Table S6 **List of significant marker trait association (MTA) identified after Bonferroni correction for different agronomical traits.** This table contains information on the entire 524 marker trait associations (MTAs) identified for different traits. Information on linked marker, F Value, p-value, phenotypic variance (PV) explained, genetic variance, residual variance and likelihood have been provided for all the MTAs.
(XLS)
