# Correction: Genomewide Association Studies for 50 Agronomic Traits in Peanut Using the ‘Reference Set’ Comprising 300 Genotypes from 48 Countries of the Semi-Arid Tropics of the World

**DOI:** 10.1371/journal.pone.0113326

**Published:** 2014-11-06

**Authors:** The PLOS ONE Staff

The legends for Figures 3 and 4 are incorrectly switched. The legend that appears for Figure 3 should be the legend for Figure 4, and the legend that appears for Figure 4 should be the legend for Figure 3. The figures appear in the correct order. Please view Figures 3 and 4 here.

**Figure 3:**
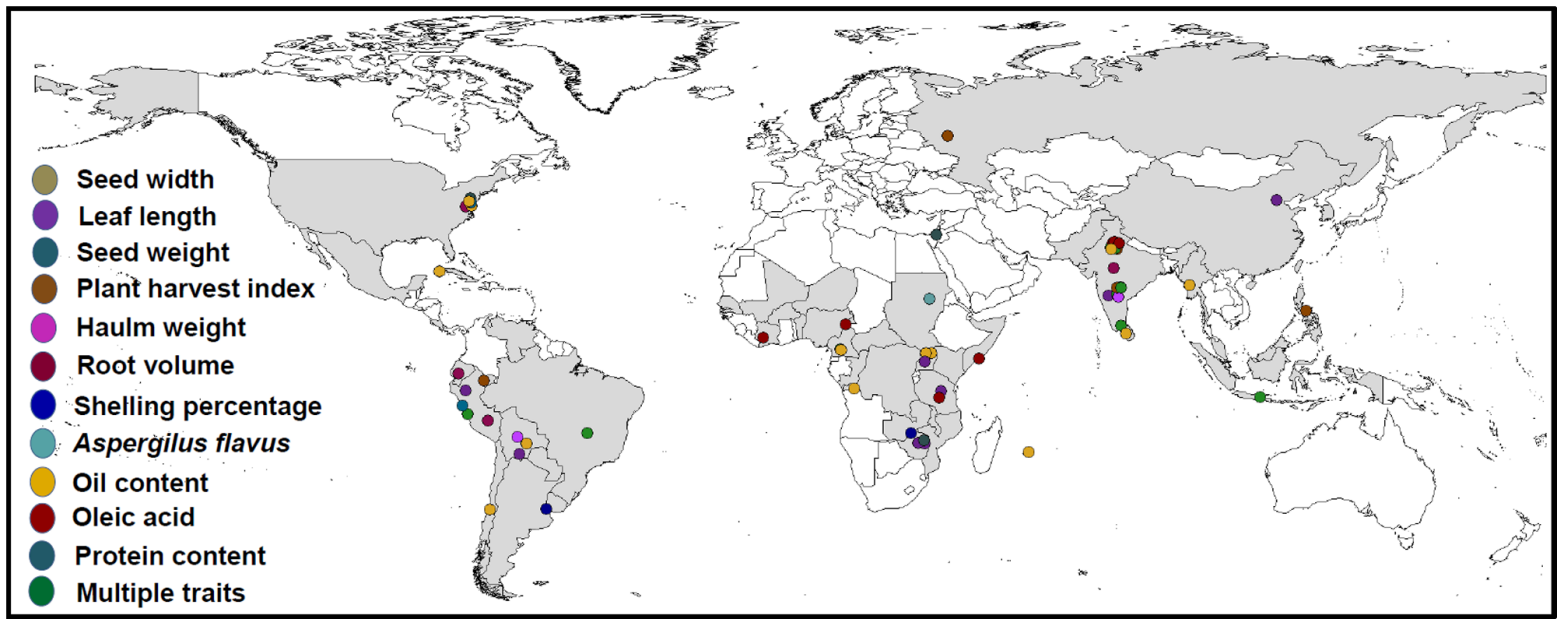
Global distribution of genotypes containing linked-marker allele(s) for different economically important traits in peanut. An attempt has been made to show passport-based geographical distribution of genotypes that had favourable alleles for markers showing association and explaining >20% phenotypic variation for the trait. Genotypes containing favourable alleles for different traits have been represented by circles in different colors.

**Figure 4:**
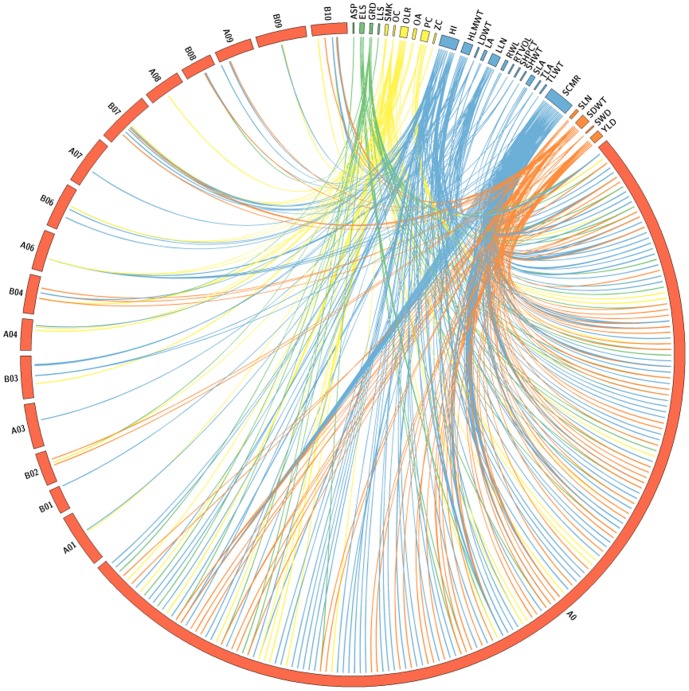
Genomewide distribution of trait-associated markers for different traits. Mapped SSR markers that showed trait association are represented on linkage groups (A01 to A10 and B01 to B10) while unmapped DArT features are assigned to A0 linkage group for representation.
